# Innovative Application of Mechanical Activation for Rare Earth Elements Recovering: Process Optimization and Mechanism Exploration

**DOI:** 10.1038/srep19961

**Published:** 2016-01-28

**Authors:** Quanyin Tan, Chao Deng, Jinhui Li

**Affiliations:** 1 State Key Joint Laboratory of Environment Simulation and Pollution Control, School of Environment, Tsinghua University, Beijing, 100084, China; 2 Basel Convention Regional Centre for Training and Technology Transfer for Asia and the Pacific, Beijing 100084, China

## Abstract

With the rapidly expanding use of fluorescent lamps (FLs) and increasing interest in conservation and sustainable utilization of critical metals such as rare earth elements (REEs), the recovering of REEs from phosphors in waste FLs is becoming a critical environmental and economic issue. To effectively recycle REEs with metallurgical methods, mechanical activation by ball milling was introduced to pretreat the waste phosphors. This current study put the emphasis on the mechanical activation and leaching processes for REEs, and explored the feasibility of the method from both theoretical and practical standpoints. Results showed physicochemical changes of structural destruction and particle size reduction after mechanical activation, leading to the easy dissolution of REEs in the activated samples. Under optimal conditions, dissolution yields of 89.4%, 93.1% and 94.6% for Tb, Eu and Y, respectively, were achieved from activated waste phosphors using hydrochloric acid as the dissolution agent. The shrinking core model proved to be the most applicable for the leaching procedure, with an apparent activation energy of 10.96 ± 2.79 kJ/mol. This novel process indicates that mechanical activation is an efficient method for recovering REEs from waste phosphors, and it has promising potential for REE recovery with low cost and high efficiency.

With the increasing focus on energy conservation, the use of fluorescent lamps (FLs) has been rapidly expanding all over the world, with the aim of phasing out incandescent lamps, because the FLs can reduce electricity consumption by at least 65%, with the same light output as for incandescent lamps, and can last up to 10 times as long^1^. The production of FLs in mainland China, the largest FL production base of the world, has increased more than 28 times in the last two decades^2^,^3^. Meanwhile, it is estimated that the amount of waste FLs generated in mainland China could reach 4.13 billion units (about 0.62 million tonnes) in 2015^4^. Managing the waste FLs in an environmentally sound way could not only mitigate the environmental and health concerns caused by the toxic mercury they contain, but also facilitate the recycling of valuable materials they also contain^5^,^6^, such as the glass, the aluminum in the caps, and several rare earth elements (REEs) in phosphors.

Phosphors are the essential components for converting ultraviolet radiation into visible light, and generally account for 2-3% of the mass of an FL^7^,^8^. Phosphor is one of the major application fields of REEs, and has a much higher content of REEs than of natural minerals. It has been shown in previous studies that REEs makes up more than 23% of the phosphors in FLs^9^,^10^,^11^, which is more than 10 and 150 times, respectively, of the minimum industrial grade of primary ores (1.5–2.0%) and ion-adsorption ores (0.08%–0.15% for light REEs, 0.06%–0.10% for heavy REEs) of REEs^12^.

Recycling metals from end-of-life products is an approach to secure their supply and achieve sustainability of metals resources^13^,^14^. At present, the recycling rate of REEs is very limited (no more than 1%)^15^, but researchers from all over the world have been intensively studying methods of recovering REEs from phosphors in FLs^5^,^16^, especially for the europium (Eu) and yttrium (Y)^17^. Supercritical liquid extraction^18^, hydrometallurgy^8^,^9^,^10^,^19^,^20^,^21^,^22^, pyrometallurgy^23^,^24^,^25^,^26^ and electrometallurgy^27^ are the approaches currently employed for REE recovery.

Taking advantage of the low viscosity and high diffusion coefficient of supercritical carbon dioxide, Shimizu *et al*.^18^ succeeded in preventing the separation of excess aqueous droplets generated during the dissolution of metal oxides, by diluting the extraction system (a complex of TBP, HNO_3_ and H_2_O) with tri-n-butyl phosphate (TBP) anhydrate. An extraction efficiency of more than 99% was achieved for Y and Eu in phosphors, but it was no more than 7% for lanthanum (La), cerium (Ce) or terbium (Tb). Hydrometallurgy is a promising alternative for metal recovery from wastes because of its adaptability to sources and its operational scale, as well as its relatively low energy consumption and cost. However, a disappointingly low recovery ratio has been obtained because the REEs in green and blue phosphors are difficult to leach out under moderate conditions^8^,^9^,^10^,^19^,^21^,^22^.

Pyrometallurgy—melting or calcination of phosphors with alkali materials—has been used by some researchers to address the leaching problem of REEs^23^,^24^,^25^,^26^, but its drawbacks of high energy consumption and reaction temperature, as well as the huge consumption of alkali material and acid required for leaching, have yet to be overcome. The electrolytic reduction method developed by Gourishankar *et al*.^27^, with the combination of molten salt and halogenation by chlorine gas, have succeeded in reducing the reaction temperature to about 500 °C; however the method still requires considerable electricity consumption for electrolytic reduction, and there is a potential for chlorine gas emission. Thus, a process yielding a high recovery ratio of REEs, with low energy consumption and low cost, would be highly desirable for waste phosphors recovery.

The mechanical activation method has shown promise for recovering metal from wastes because it triggers physicochemical changes from particle comminution, new surface generation, crystalline structure defects, polymorphic transformations, and even direct reactions^28^,^29^. In this study, we have developed a novel process to recover REEs from waste phosphors by enhancing leaching efficiency with mechanical activation. In order to systematically and completely develop the REE recovery process from waste phosphors, this work was devoted to finding the optimal leaching process and to investigate its mechanism with experiments and applicable theory. Obtained results and findings were expected to contribute to a closed-loop process for FLs and sustainable development for REE industries.

## Experimental Section

### Chemicals and materials

A mixture of waste phosphors and cullet was obtained from the Beijing Eco-island Science and Technology Co., Ltd.; this mixture represented the waste phosphors fraction output from a waste FL treatment plant. The mixture was sieved with a 300-mesh screen to remove most of the cullet, and collected as a waste phosphors sample for use in the experiments. All reagents were purchased from Beijing Chemical Works and were of analytical grade.

### Mechanical activating operation

The waste phosphors sample was mechanically activated using a planetary ball-mill apparatus (P-7, Fritsch, Germany) under an ambient atmosphere. In each batch of activation, 5 g of the waste phosphors sample was put into a zirconia pot (45 mL inner volume) with 7 zirconia balls (15 mm in diameter), and the mill was run and paused alternately for 15-min intervals, to avoid generating too much heat. All the activated samples were subjected to leaching after the mechanical activation, with no further treatment.

### Experimental design

The Plackett-Burman (PB) experimental design approach is one of the most commonly used two-level experimental designs^30^,^31^, which was adopted to determine the acid (sulfuric, hydrochloric, or nitric) with the best performance for leaching REEs from the activated samples, and to evaluate the individual significance of each factor. An orthogonal experiment design approach was adopted to optimize the process.

### Characterization of the test specimens

The samples and specimen had been fully characterized by Inductively Coupled Plasma-Atomic Emission Spectroscopy (ICP-AES) and ICP-MS, as well as by X-ray-based techniques such as X-ray Fluorescence (XRF), X-ray Diffraction (XRD) and Scanning Electron Microscopy interfaced with X-ray microanalysis (SEM-EDS).

#### ICP-AES and ICP-MS characterization

Aliquots of 0.3 g were subsampled from the waste phosphors sample, then introduced in polyfluoroalkoxy (PFA) vessels containing 16 mL aqua regia and subjected to microwave-assisted acid digestion (MDS-8, Sineo, China). A blank specimen was prepared using the same program without the addition of a waste phosphors sample. The REEs were determined using an ICP-AES (ThermoFisher IRIS Intrepid II XSP), and an ICP-MS (ThermoFisher X Series 2) for elements with a concentration below the determination limit of ICP-AES. Three independent experiments were conducted to determine the average values of REE content; results are reported in Table 1. Six independent spike and recovery tests were conducted; results were in the range of 95%–105%.

#### Morphological, dimensional and compositional analysis

Morphological characterizations of the samples were obtained with SEM-EDX (Merlin Compact, Zeiss, Germany). The particle sizes and specific surface areas of the waste phosphors samples before and after the mechanical activation were individually analyzed using a laser diffraction particle size analyzer (Malvern Mastersizer 2000, UK) and the BET method (Tristar II 3020, Micromeritics Instrument Corporation, USA). Compositional information had been collected, on representative aliquots of the sample, using XRF (XRF-1800, Shimadzu, Japan). The mineralogical analysis of the sample was investigated with XRD (D8 Advance, Bruker, Germany).

## Results and Discussion

### Chemical composition of waste phosphors

The chemical composition of the waste phosphors was analyzed with XRF, as well as with microwave-assisted aqua regia digestion and ICP-AES, and is presented in Table 1. REEs such as Y, Eu, La, Ce, and Tb existed in concentrations of approximately 40%, while the other main elements in the waste phosphors sample were found to be oxygen (O), silicon (Si), aluminum (Al), phosphorus (P), and calcium (Ca).

The monthly prices of REEs from January 2014 to June 2015 released by the Association of China Rare Earth Industry were collected. The relative values of the five REEs in phosphors were compared with the combined percentages of their content in phosphors. Tb, Eu, and Y accounted for 58.6%, 32.2%, and 7.4%, respectively (98.13% in total), of the total value of the REEs in phosphors. Therefore, when trying to optimize the activation and leaching processes, the emphasis was put on Tb, Eu, and Y. Then, for exploring the leaching kinetics, efforts were focused on Tb.

### Acid selection by means of Plackett-Burman experiments

Before the leaching tests, preliminary tests were conducted on both inactivated and activated (700 rpm, 120 min) phosphors using H_2_SO_4_, HCl, and HNO_3_. After leaching for 3 hours at 70 °C, significant improvements in the leaching rates of Tb, Ce, and La could be observed for the activated samples: more than 80%, 65%, and 80%, respectively, compared with less than 3% for the inactivated samples. Meanwhile, the average leaching rate of Tb in activated waste phosphors with H_2_SO_4_ was 50.6%, and it averaged 80.5% and 80.9%, respectively, with HCl and HNO_3_. No differences were observed in the leaching rates of Eu or Y for either the inactivated or the activated waste phosphors, with any acid.

No consistent conclusion has been obtained in previous studies, either, on the performance of different acids (H_2_SO_4_, HCl, HNO_3_) when used for leaching REEs from inactivated waste phosphors^5^. Moreover, these preliminary tests were conducted under a few specific conditions, limiting the applicability of the results. Therefore, the acid to be used for further study was determined via PB experiments (details of the experiment design and results can be found in Table S1 in the supplementary information (SI), the matrix for the design could be found in ref. 30).

**Figure 1:**
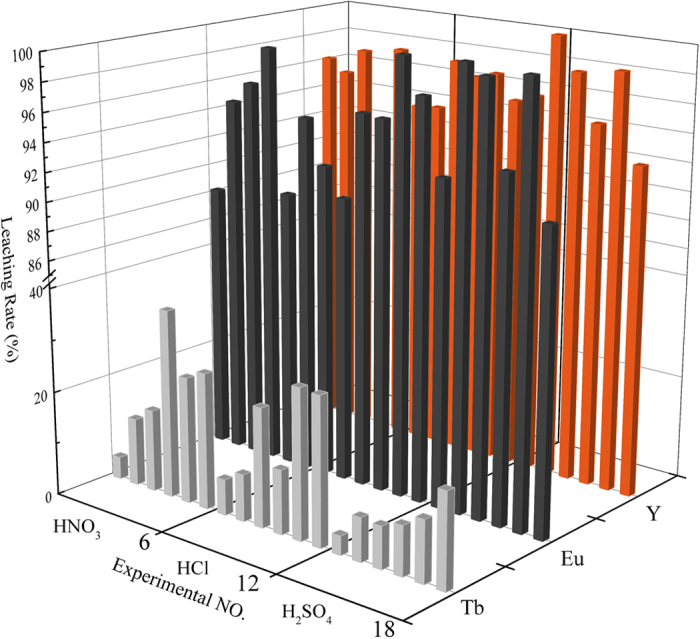
Results of PB experiments using different acids.

The results shows that no differences were observed in the leaching rates of Eu or Y with these three acids, and this is similar as conclusion obtained in the preliminary tests. The average leaching rate of Eu and Y by the three acids were ranged in 93.70%–95.91%, and 94.09%–96.75%, respectively. As for the leaching of Tb, HCl and HNO_3_ showed similar leaching efficiencies for activated waste phosphors with the average rate of 18.06% and 19.95%, respectively; but an obvious reduction of about 10% the leaching rate could be noted when H_2_SO_4_ was used (Fig. 1). In this study, HCl was preferred over HNO_3_ because of its lower cost and its leaching rate for the three REEs. Based on these results, PB experiments using only HCl (SI Table S1) were conducted to primarily explore the significance of each factor, and the results suggested that rotational speed, acid concentration, and liquid-solid ratio exerted the most influence, followed by milling time, reaction temperature, and leaching time.

### Optimizing the activation and leaching process for REEs

The orthogonal experiment method was adopted for experiment design, to optimize conditions for high REE leaching rates. The results and analyses are shown in Tables 2 and 3. It was demonstrated that the optimal conditions for the processes of activation and leaching as a whole were: milling at a rotational speed of 600 rpm for 60 min, using 6 M HCl for leaching at the liquid-solid ratio of 60 (mL/g), with a leaching time of 15 min at 60 °C. The extreme differences in analysis results (Table 3) demonstrated that the factors that contribute most to the activation and leaching processes, using acid concentration as the first priority, were: liquid-solid ratio, rotational speed, milling time, reaction temperature, and leaching time—the same sequence as was obtained by the PB experiments, except for rotational speed. This one difference could be explained in that the PB experiments were designed to do a preliminary screening of factors under a narrower scope, with fewer test runs^32^.

**Figure 2:**
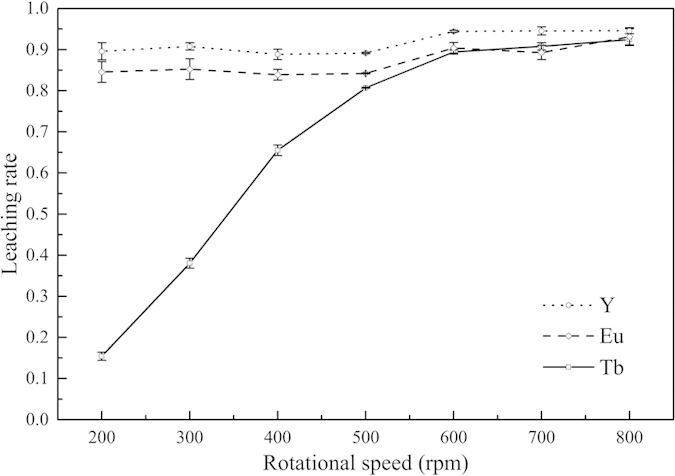
Leaching rate of selected REEs Tb, Eu, and Y dissolved from phosphors activated at different rotational speeds. Error bars represent the standard error of the mean for three replicates.

Figure 2 represents the leaching rates of the three selected REEs: Tb, Eu, and Y, dissolved from the phosphors and activated at different rotational speeds. The results illustrate that the rotational speed had a positive effect on the leaching rate of Tb; it ranged from 15.4% to 89.4% when the rotational speed increased from 200 rpm to 600 rpm, then showed a slight improvement (3.1%) with the increase of rotational speed from 600 rpm to 800 rpm. Most of the Tb was leached out from a practically insoluble state on the phosphate and/or aluminate side. This result also indicates that the local structure of the phosphors was altered via mechanical activation.

As for the leaching rates of Eu and Y, no significant relationship was observed between these rates and rotational speed. The leaching rates fluctuated with the rotational speed in the ranges of 83.9%–93.1% and 88.9%–94.6%, respectively. Meanwhile, the detailed leaching results under different acid concentrations and liquid-solid ratios are presented in SI Figs S1 and S2, which further support the previously determined optimal conditions.

The method showed similar leaching rates for Eu and Y, to those obtained in studies using the conventional hydrometallurgical method: around 95% after optimization of conditions^5^—rates that also matched results from a study employing the alkali sintering method, by Liu *et al*.^33^, which achieved the highest rates: 99.05% and 94.6% for Eu and Y, respectively. A high leaching rate was achieved for Tb, compared to those obtained by conventional methods (generally around 10%); in addition, an improvement of 13.2% was achieved for the leaching rate of Tb in this study, compared with the study by Liu *et al*.^33^

### Exploration of mechanisms for the leaching process

Experiments under different temperatures, in the same range as in the orthogonal experiments, were conducted to uncover the mechanism of the REE leaching process from activated waste phosphors. At the initial stage of leaching, in all the temperature scenarios studied, the leaching rates (concentration in solution) increased rapidly with an increase in leaching time, but the increase gradually slowed down and eventually stabilized. Meanwhile, an increase in the leaching temperature enhanced the leaching process, raising the maximum leaching rate and reducing the leaching time.

For the investigation of REE leaching kinetics, the previously established shrinking core model played an important role in the fluid-solid systems^34^,^35^,^36^. Therefore, the shrinking core model was adopted to describe the leaching kinetics. The equations for this model can be expressed as follows.


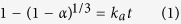



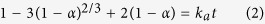


where *α* is the leaching rate of the studied element at time *t* (min), and *k*_*a*_ represents the apparent reaction rate constant (min^−1^). Eq. (1) describes the leaching process when the solid particles are of a spherical geometry and the chemical reaction is the rate-controlling step, while Eq. (2) considers that the diffusion of the reagent through the boundary layer is the rate-controlling step.

**Figure 3:**
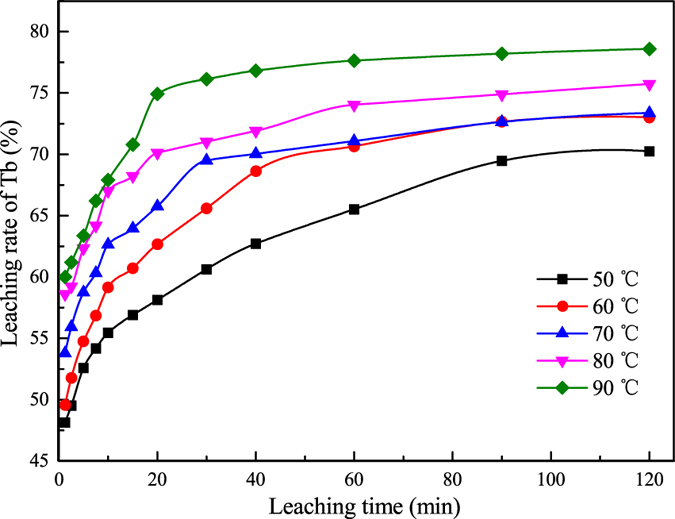
Leaching rates of Tb under different temperatures.

**Figure 4:**
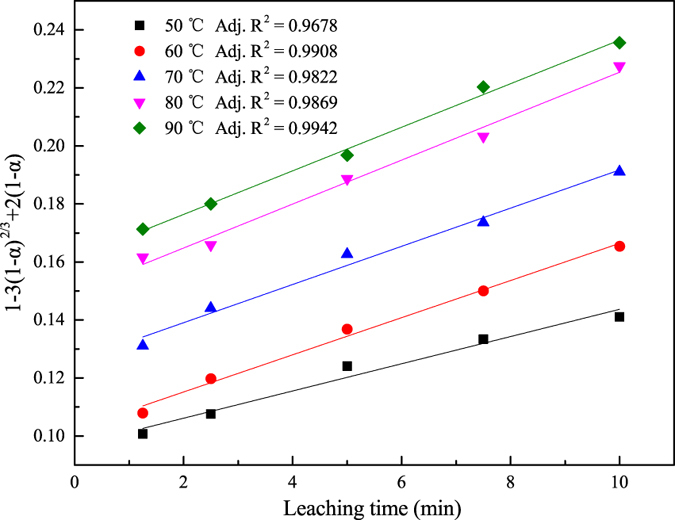
Plots of [1 − 3(1 − α)^2/3^ + 2(1 − α)] versus time at various leaching temperatures for 60 min, for activated waste phosphors.

As demonstrated in the Fig. 3 that the leaching temperature shows positive influence to the leaching rate of Tb under the experiment condition. Meanwhile, the leaching process will reach equilibrium with the prolonging of the leaching time and the leaching rate of Tb does not obviously increase after the equilibrium what is closely related to the leaching temperature. The plots of [1 − 3(1 − *α*)^2/3^ + 2(1 − *α*)] versus leaching time using data of Fig. 3 are presented in Fig. 4. The value of the adjusted coefficient of determination (Adj. R^2^) reveals that Eq. (2) fits the data best, and the minimum value of the adjusted coefficient of determination (Adj. R^2^) is 0.9678. The results suggest that the leaching of activated waste phosphors is controlled by the diffusion step.

On the basis of the apparent reaction rate constant (*k*_*a*_) from Fig. 4, the Arrhenius equation (Eq. (3)) was employed to determine the activation energy.


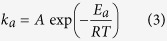


where *A* represents the frequency factor (min^−1^); *R* is the universal gas constant (8.314 J·K^−1^·mol^−1^); *T* is the reaction temperature (K); and *E*_*a*_ represents the apparent activation energy (kJ/mol).

A straight line between ln*k*_*a*_ and 1/*T* was obtained with the adjusted coefficient of determination (Adj. R^2^) of 0.7835, and the apparent activation energy (*E*_*a*_) for the activated waste phosphors was 10.96 ± 2.79 kJ/mol, and it was the same magnitude as that of the absorption of Eu (III) in solution by graphene oxide nanosheets (7.14 kJ/mol)^37^, which shows the leaching reaction was easy to happen. In general, the apparent activation energy of the diffusion-controlled leaching is ~20 kJ/mol; it is >40 kJ/mol for a chemical reaction-controlled leaching^38^,^39^,^40^. The value of the apparent activation energy further supported the result that the rate-controlling step of the leaching process of activated waste phosphors was the diffusion of the reagent through the boundary layer.

Notwithstanding, a good fit of [1− (1−*α*)^1/3^] versus time could also be achieved based on the data presented in Fig. 3. An apparent activation energy of only 6.00 kJ/mol (<20 kJ/mol) was determined with the Arrhenius equation with the apparent reaction rate constant (*k*_*a*_) obtained at different temperatures. Clearly, the chemical reaction was not the rate-controlling step.

The apparent reaction order (*n*) of the leaching process with respect to HCl was determined based on experiments conducted using different concentrations of HCl ([HCl]), including 0.2, 0.4, 0.5, 0.8, and 1.0 M, at the same temperature (60 °C). Therefore, the relationship between the apparent reaction order and the HCl concentration could be simplified and expressed as follows:


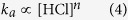


As already stated, the *k*_*a*_ was calculated using Eq. (2) and the same method as for Fig. 4. Good fittings of [1 − 3(1 − *α*)^2/3^ + 2(1 − *α*)] versus time were observed, and the minimum value of the adjusted coefficient of determination (Adj. R^2^) was 0.8017. After that, a fit with the adjusted coefficient of determination (Adj. R^2^) of 0.9214 was achieved for the linear relationship of ln*k*_*a*_ versus ln[HCl], and the determined apparent reaction order was 1.16 ± 0.17.

After the investigation of leaching kinetics of Tb from inactivated waste phosphors, the leaching of Tb appeared to be a chemical reaction-controlled process. This suggestion was further supported by the result of the apparent activation energy, which was determined to be 52.82 ± 3.95 kJ/mol. The apparent reaction order for the leaching process was 0.67 ± 0.06. The results indicate that the mechanical activation process could cause destruction of the crystal structure of the phosphors, which could be the reason for the sharp decrease in apparent activation energy, leading to the transformation of the rate-controlling step from chemical reaction to diffusion, and making the REE leaching much easier^41^,^42^.

### Analysis of the physicochemical changes in prepared samples after leaching

In order to further explore the mechanical activation process of phosphors, physicochemical changes were also investigated in detail, for inactivated and activated waste phosphors, and for the leaching residues. The activated waste phosphor samples were collected after milling for 60 min at the rotational speed of 600 rpm, while the leached residues were washed with deionized water after filtration, then dried for further measurements.

**Figure 5:**
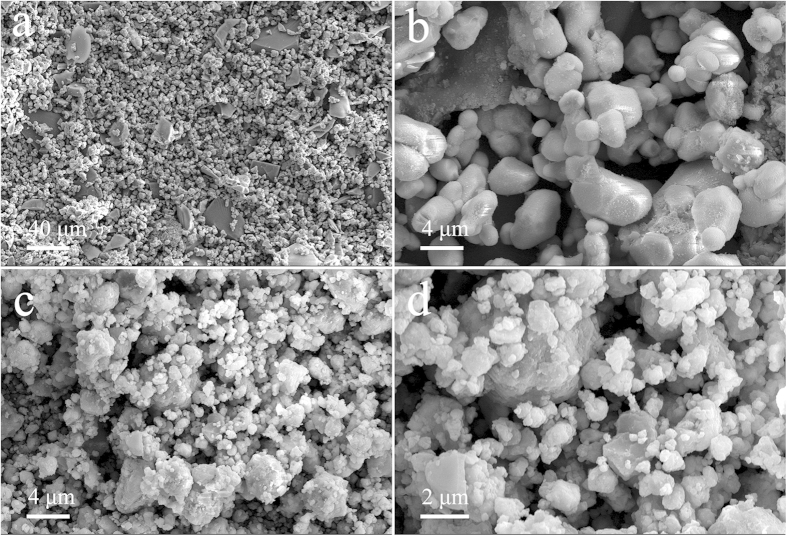
SEM images of inactivated and activated (600 rpm, 60 min) waste phosphors: **(a)** inactivated, ×300; **(b)** inactivated, ×3,000; **(c)** activated, ×3,000; **(d)** activated, ×7,000.

As presented in the SEM images of inactivated waste phosphors (Fig. 5), glass particles, which had relatively larger particle sizes, were mixed in with the waste phosphors. A significant reduction in the size of the particles could be observed after the activation process. There was an obvious increase in the amount of fine particles (with particle sizes below 2 μm) in the activated waste phosphors. This result was well consistent with the determination of particle size distribution. After activation, the median particle size (D_50_) of the particles was reduced to 7.20 μm from 20.65 μm in the inactivated waste phosphors, and the minimum particle size was reduced from about 2 μm to about 30 nm.

It were also demonstrated by the SEM images that the surfaces of the particles became rougher in the inactivated waste phosphors after activation, a result that was also supported by the specific surface area tests. The specific surface area increased to 1.82 m^2^/g after activation, which was 13 times that of the inactivated waste phosphors (0.14 m^2^/g). A slight aggregation of the particles was observed after activation for 60 min under 600 rpm. Besides, no obvious pores could be observed, according to the surface morphology of the waste phosphors, which further supported the utilization of the shrinking core model (for non-porous solids) rather than the models for porous solids, such as the random pore model, the uniform pore model or the grain model, for the analysis of experimental data, as mentioned previously.

Although the reduction of particle sizes and the increase in specific surface area of particles could improve the leaching process, a change in the crystal structure of particles should be the main reason for the significant reduction of the apparent activation energy of the leaching process, and this result was confirmed by XRD tests on the inactivated and activated waste phosphors.

**Figure 6:**
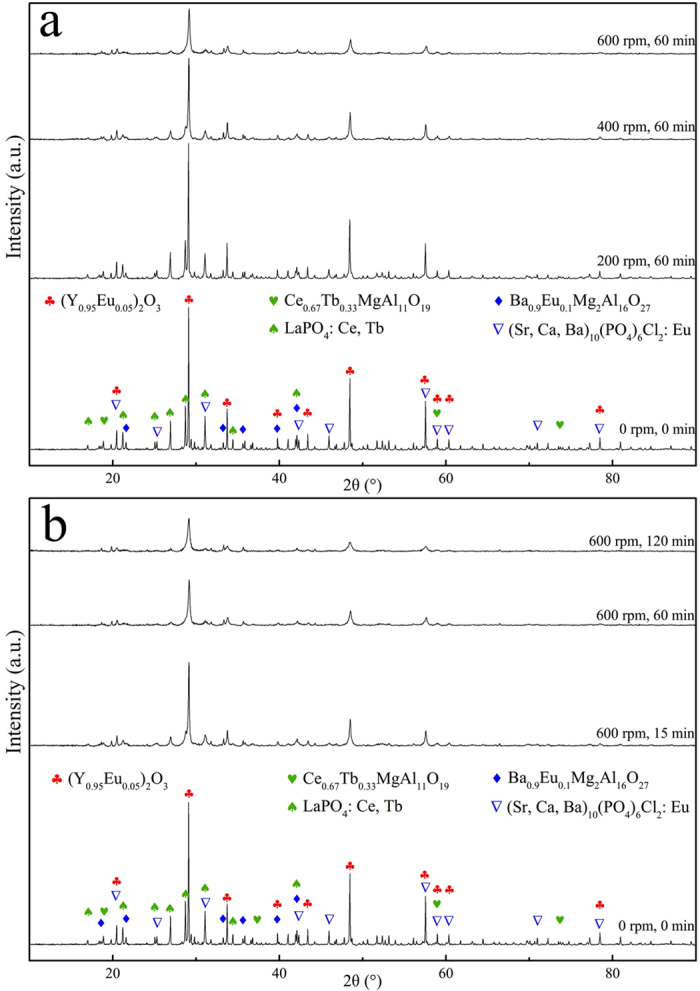
XRD patterns of inactivated and activated waste phosphors.

According to the XRD pattern results, which has been presented in Fig. 6, the crystal structure changed dramatically after mechanical activation. The XRD pattern intensity decreased with both the increase of rotational speed and milling time. When the mechanical activation was conducted under the condition of 600 rpm and 60 min, the intensity had decreased to about 1/3 of that of inactivated sample. Besides, the full width at half-maximum (FWHM) of peaks also broaden with the increase of rotational speed and milling time. It shows that the crystal structure of the particles was destroyed and gradually transformed to a disordered state due to the friction and impact during activation^43^. Meanwhile, the internal stress of crystal lattice would also increase with the progress of activation^44^. These changes could cause the reduction of activation energy for leaching reaction^43^,^44^.

## Conclusions

In this study, an efficient and sustainable method for the recovery of REEs from waste phosphors in FLs has been proposed, in combination with an innovative application of mechanical activation. Mechanical activation plays a crucial role in the developed method, and it has been successfully exploited for the dissolution of REEs under moderate conditions, requiring less energy and materials consumption.

The experimental results indicate that HCl presents a better performance when compared with HNO_3_ and H_2_SO_4_, and that rotational speed, acid concentration, and liquid-solid ratio significantly influence the REE extraction. After optimization of conditions, high dissolution yields of 89.4%, 93.1% and 94.6% for Tb, Eu and Y, respectively, were achieved, from activated waste phosphors using hydrochloric acid. Moreover, the shrinking core model strongly suggests that the rate-determining step of the extraction procedure changed from the chemical reaction to diffusion, after mechanical activation, with apparent activation energy reduced from 52.82 ± 3.95 kJ/mol to 10.96 ± 2.79 kJ/mol. The proposed technique offers the benefits of low cost and high efficiency, and reveals a high feasibility for recovering REEs from waste phosphors. It can be expected to contribute to a closed loop of REE resources utilization, from the perspectives of both the environment and the economy, in accordance with the concept of a circular economy.

## Additional Information

**How to cite this article**: Tan, Q. *et al*. Innovative Application of Mechanical Activation for Rare Earth Elements Recovering: Process Optimization and Mechanism Exploration. *Sci. Rep.* **6**, 19961; doi: 10.1038/srep19961 (2016).

## Supplementary Material

Supplementary Information

## Figures and Tables

**Table 1:** Chemical composition of waste phosphors.

**REEs**				**Other elements**			
**Element**	**Mass (wt%)**	**Element**	**Mass (wt%)**	**Element**	**Mass (wt%)**	**Element**	**Mass (wt%)**
Y	21.33	O	32.01	Na	2.08	K	0.40
La	10.40	Si	9.09	Ba	1.86	Cl	0.10
Ce	4.64	Al	4.21	Mg	0.83	S	0.02
Tb	2.49	P	4.00	Sr	0.80	Zn	0.02
Eu	1.55	Ca	3.71	Fe	0.46		

**Table 2:** Experiment results using orthogonal design.

**Run**	**Rotational speed (rpm)**	**Milling time (min)**	**Acid concentration (M)**	**Reaction temperature (°C)**	**Leaching time (min)**	**Liquid-solid ratio (mL/g)**	**R**_**Tb**_ **(%)**	**R**_**Eu**_ **(%)**	**R**_**Y**_ **(%)**
1	200	15	0.5	50	15	10	0.93	18.84	19.03
2	200	30	1	60	30	20	1.55	82.66	85.14
3	200	60	2	70	60	40	10.43	95.14	98.41
4	200	120	4	80	120	60	73.02	91.05	99.35
5	200	240	6	90	240	120	97.51	88.89	97.70
6	300	15	4	60	60	120	30.24	89.67	98.74
7	300	30	6	70	120	10	34.17	91.88	95.28
8	300	60	0.5	80	240	20	5.86	61.85	87.01
9	300	120	1	90	15	40	20.54	93.64	97.57
10	300	240	2	50	30	60	53.66	77.28	83.25
11	400	15	1	70	240	60	17.97	86.58	97.52
12	400	30	2	80	15	120	55.32	88.43	97.33
13	400	60	4	90	30	10	42.07	91.72	95.62
14	400	120	6	50	60	20	75.18	93.15	95.58
15	400	240	0.5	60	120	40	46.47	84.94	96.65
16	500	15	6	80	60	40	81.88	89.37	97.42
17	500	30	0.5	90	60	60	23.77	91.34	98.10
18	500	60	1	50	120	120	59.11	88.13	97.44
19	500	120	2	60	240	10	48.58	83.80	93.94
20	500	240	4	70	15	20	85.50	93.22	95.36
21	600	15	2	90	120	20	32.42	91.04	95.47
22	600	30	4	50	240	40	87.12	90.97	97.61
23	600	60	6	60	15	60	97.31	91.76	95.16
24	600	120	0.5	70	30	120	64.53	85.70	97.18
25	600	240	1	80	60	10	33.31	39.55	68.27

**Table 3:** Factor levels and results analysis for orthogonal design.

**Level**		**Rotational speed (rpm)**	**Milling time (min)**	**Acid concentration (M)**	**Reaction temperature (°C)**	**Leaching time (min)**	**Liquid-solid ratio (mL/g)**
1		200	15	0.5	50	15	10
2		300	30	1	60	30	20
3		400	60	2	70	60	40
4		500	120	4	80	120	60
5		600	240	6	90	240	120
Extreme difference (%)	Tb	34.04	30.60	50.71	12.68	11.47	29.53
	Eu	13.85	14.37	22.79	17.65	12.23	25.65
	Y	16.61	15.09	17.74	18.31	15.95	23.25
Priority order	Acid concentration >liquid-solid ratio >rotational speed >milling time >reaction temperature >leaching time						
Optimal level		600	60	6	60	15	60
