# Supplementary material for: Innovative Application of Mechanical Activation for Rare Earth Elements Recovering: Process Optimization and Mechanism Exploration
Source: Sci Rep. 2016 Jan 28;6:19961. doi: 10.1038/srep19961 (PMC4730208; doi:10.1038/srep19961)
Supplement: Supplementary Information [file srep19961-s1.pdf]

Supplementary Information

Manuscript Title:

Innovative Application of Mechanical Activation for Rare Earth Elements Recovering: Process Optimization and Mechanism Exploration

Quanyin Tan [a], Chao Deng [b], Jinhui Li [a] *

[a] State Key Joint Laboratory of Environment Simulation and Pollution Control, School of Environment, Tsinghua University, Beijing, 100084, China

[b] Basel Convention Regional Centre for Training and Technology Transfer for Asia and the Pacific, Beijing 100084, China

Table S1 Experiments results in given Plackett-Burman design

NO	Milling condition		Leaching condition					Leaching rate (%)		
	Rotation speed (rpm)	Milling Time (min)	Proton concentration (mol/L)	Temperature (℃)	Retention time (min)	Liquid-solid ratio (mL·g $^{-1}$)	Acid	Tb	Eu	Y
1	300	30	1	60	30	20	HNO_3	4.29	88.28	84.84
2	300	30	2	60	60	40		13.02	94.88	96.64
3	300	60	2	70	30	20		15.80	96.36	95.79
4	400	30	2	70	60	20		36.17	99.03	97.55
5	400	60	1	60	30	40		24.27	89.04	91.72
6	400	60	1	70	60	40		26.16	94.62	98.00

7	300	30	1	70	30	40	HCl	6.90	91.53	94.16
8	300	60	1	70	60	20		9.06	89.55	94.28
9	300	60	2	60	60	40		22.88	95.63	97.75
10	400	30	1	60	60	20		12.31	95.47	96.85
11	400	30	2	70	30	40		28.76	100.46	97.28
12	400	60	2	60	30	20		28.43	97.42	95.61
13	300	30	1	70	30	40	H_2SO_4	3.68	92.27	96.16
14	300	60	1	70	60	20		8.38	100.02	100.48
15	300	60	2	60	60	40		8.08	99.31	98.20
16	400	30	1	60	60	20		9.48	93.47	94.92
17	400	30	2	70	30	40		11.84	99.78	98.65
18	400	60	2	60	30	20		18.17	90.65	92.60
19	300	30	1	60	30	20	HCl	4.85	88.74	88.23
20	300	30	2	60	60	40		13.90	94.08	96.23
21	300	60	2	70	30	20		7.54	99.52	97.93
22	400	30	2	70	60	20		19.09	97.67	97.35
23	400	60	1	60	30	40		25.41	91.80	95.54
24	400	60	1	70	60	40		25.80	93.38	98.08

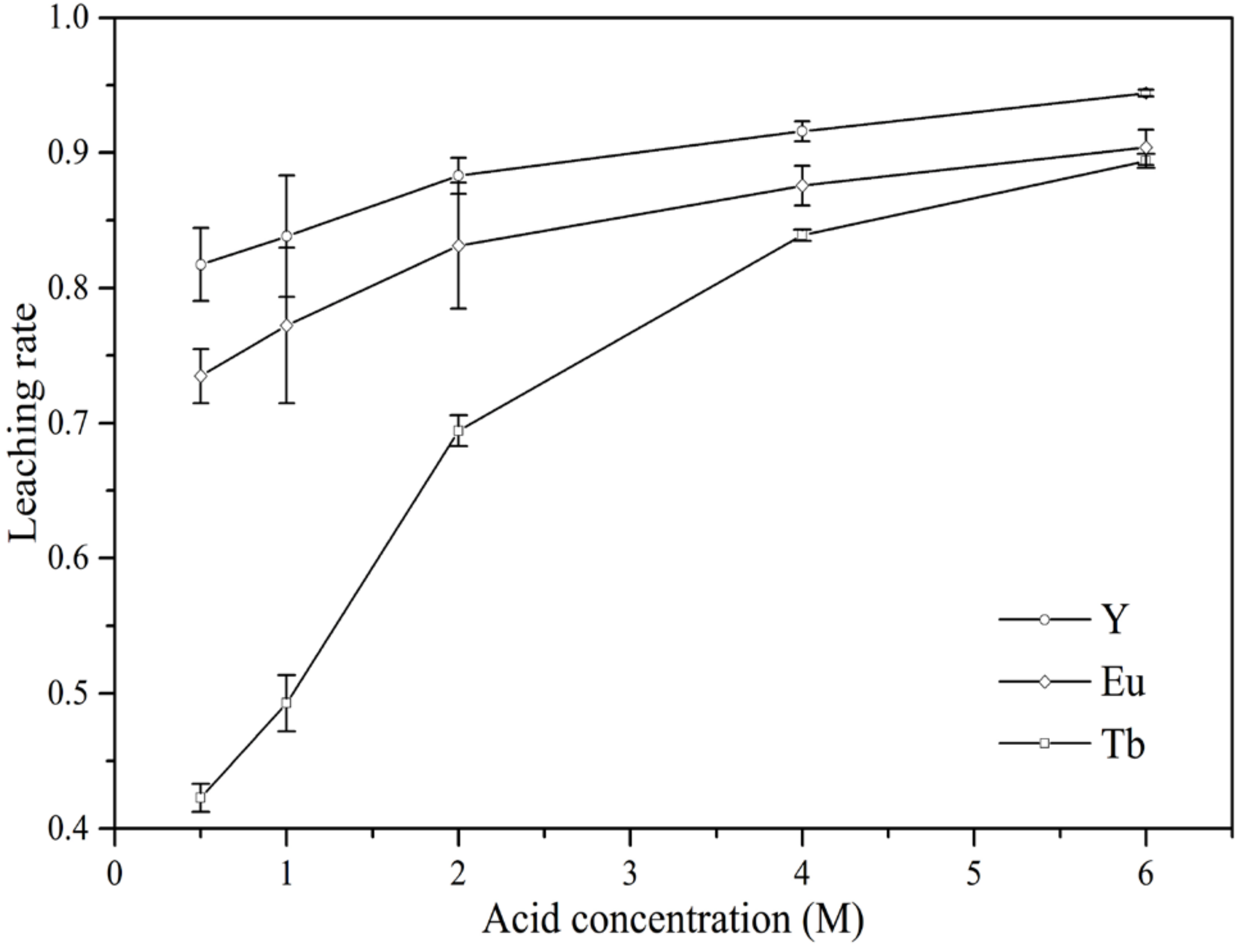

Figure S1 Leaching rate of selected REEs Tb, Eu, and Y dissolved from activated phosphors in different acid concentration

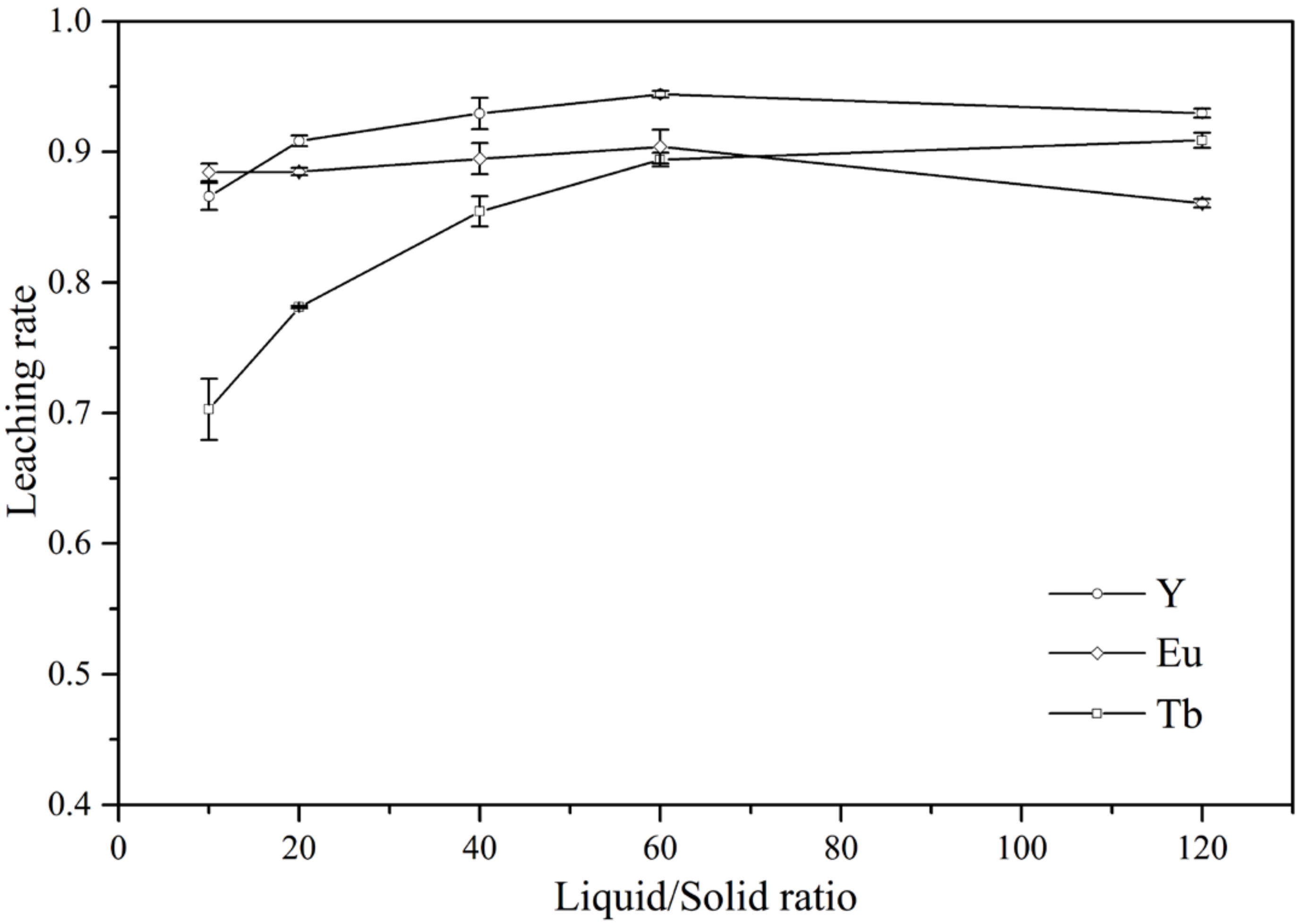

Figure S2 Leaching rate of selected REEs Tb, Eu, and Y dissolved from activated phosphors in different liquid-solid ratio
